# Development of Stable Pickering Emulsions with TEMPO-Oxidized Chitin Nanocrystals for Encapsulation of Quercetin

**DOI:** 10.3390/foods12020367

**Published:** 2023-01-12

**Authors:** Xiaoxue Jia, Peihua Ma, Kim Shi-Yun Taylor, Kevin Tarwa, Yimin Mao, Qin Wang

**Affiliations:** 1 Department of Nutrition and Food Science, College of Agriculture and Natural Resources, University of Maryland, College Park, MD 20742, USA; 2 Department of Materials Science and Engineering, University of Maryland, College Park, MD 20742, USA; 3 NIST Center for Neutron Research, National Institute of Standards and Technology, Gaithersburg, MD 20899, USA

**Keywords:** chitin nanocrystals, TEMPO, Pickering emulsion, quercetin, stability

## Abstract

Pickering emulsions stabilized by TEMPO-oxidized chitin nanocrystals (T-ChNCs) were developed for quercetin delivery. T-ChNCs were synthesized by TEMPO oxidation chitin and systematically characterized in terms of their physicochemical properties. T-ChNCs were rod-like with a length of 279.7 ± 11.5 nm and zeta potential around −56.1 ± 1.6 mV. The Pickering emulsions were analyzed through an optical microscope and CLSM. The results showed that the emulsion had a small droplet size (972.9 ± 86.0 to 1322.3 ± 447.7 nm), a high absolute zeta potential value (−48.2 ± 0.8 to −52.9 ± 1.9 mV) and a high encapsulation efficiency (quercetin: 79.6%). The emulsion stability was measured at different levels of T-ChNCs and pH values. The droplet size and zeta potential decreased with longer storage periods. The emulsions formed by T-ChNCs retarded the release of quercetin at half rate of that of the quercetin ethanol solution. These findings indicated that T-ChNCs are a promising candidate for effectively stabilizing Pickering emulsions and controlling release of quercetin.

## 1. Introduction

Quercetin (Q) is a flavonoid with potential therapeutic properties. Its anti-inflammatory, anti-tumor, neuroprotective, anti-allergic, antioxidant and antibacterial properties make it a promising nutraceutical drug for health promotion. However, the poor water solubility of quercetin, inactive metabolites and high metabolic and clearance rates make quercetin less bioavailable [1]. Therefore, the development of delivery systems for encapsulated quercetin with high bioavailability contributes to human health.

Pickering emulsions are considered as ideal systems for the encapsulation of nutraceuticals. A Pickering emulsion is an immiscible mixture of two phases stabilized by solid colloidal particles. These solid particles, whether inorganic or organic, localize at the interface of the two phases due to their surface wettability [2]. Commonly used solid particles include clays [3], silica [4], TiO_2_ [5], magnetic particles (Fe_3_O_4_) [6], hydroxyapatite [7], carbon nanotubes [8], latexes [9], polystyrene particles [10], cellulose [11], chitosan [12], cyclodextrin [13] and some food-grade solid particles such as starch, zein, soy protein and whey protein [14]. These solid particles can create an irreversible steric barrier at the oil–water interface, hindering droplet coalescence and Ostwald ripening. Electrostatic repulsion induced by charged particles also favors the stabilization procedure. However, the practical applicability of these inorganic and synthetic organic materials in Pickering emulsions is considerably limited, notably in foods and bio-related goods, due to their high cost, limited biocompatibility, advanced synthetic processes and even toxicity [15]. Recently, investigations of polysaccharide-based particles (e.g., cellulose, chitin, chitosan, starch) for the stabilization of Pickering emulsions have attracted researchers’ interest due to numerous benefits, including large quantity and high accessibility, low-cost, favorable biocompatibility and biodegradability and non-toxicity [16].

Chitin is a natural linear polysaccharide, mainly 2-acetoamido-2-deoxy-d-glucose repeating units linked by β-(1 → 4). It is the second most abundant biopolymer in the natural world after cellulose. It widely exists in marine creatures such as shrimp, crab shells and squid pens. A massive amount of waste shells makes it an abundant, cheap and easily accessible resource. However, natural chitin is a highly crystalline biopolymer with poor solubility in water and common organic solvents, limiting its direct applicability. Typically, chitin is transformed at high temperatures by deacetylation reaction with concentrated bases to its acid-soluble derivative, chitosan, a product that has been widely employed in food fields. Indeed, the unique properties of chitin, including non-toxicity, biocompatibility, biodegradability, chemical stability and antimicrobial properties, make it an ideal candidate for stabilizing emulsions.

The superior emulsion stabilization abilities of chitin particles and chitin nanocrystals (ChNCs) were demonstrated [17]. Therein, addition of high concentrations of chitin particles (2.5% *w*/*w*) achieved stable oil-in-water (O/W) emulsions [18], while the same stability was realized by reducing the chitin concentration to 0.5% w/w with the aid of 0.005% *w*/*w* Tween 80. Similarly, emulsions with a high amount of ChNCs (1.0% *w*/*w*) had an excellent stability without creaming. Since then, many studies have reported the emulsification potential of chitin nanocrystals to produce stable O/W Pickering emulsions. ChNCs were obtained from hydrogen chloride hydrolysis of shrimp shells and were employed to stabilize O/W Pickering emulsions at 1.0 and 1.5% *w*/*v* content [19]. Moreover, ChNCs created gel-like high internal phase emulsions (HIPE) with internal phase proportions reaching up to 96%, which proved to be very stable and tunable from liquid to gel texture [20]. However, ChNCs-stabilized O/W emulsions always have droplet diameters up to 100 μm, leading to a low physical stability. It has been shown that emulsions with smaller particle sizes or sufficient charge were more stable [21]. Commonly used as a reagent for oxidation, 2,2,6,6-tetramethylpiperidin-1-oxyl (TEMPO) is an organic radical. The chitin nanocrystals (T-ChNCs) obtained by TEMPO oxidation are highly negatively charged and expected to be a promising candidate for Pickering emulsion stabilization.

Thus, the objectives of the present work were: (1) to prepare T-ChNCs and characterize their physicochemical properties by Fourier-transform infrared (FTIR) and scanning electron microscopy (SEM), particle size analyzer and zeta potential; (2) to investigate the capability of T-ChNCs to stabilize O/W emulsions. Droplet size of emulsions was measured and zeta potential was obtained by measuring electrophoretic mobility, the emulsion microstructure was observed by confocal laser scanning microscope (CLSM) and the changes in droplet size and zeta potential were determined; (3) to develop a delivery system for quercetin using T-ChNCs as the stabilizer. Encapsulation efficiency, antioxidant activities and quercetin release profiles were determined.

## 2. Materials and Methods

### 2.1. Chemicals and Reagents

Chitin from shrimp shells was obtained from Sigma-Aldrich (St Louis, MO, USA) and purified before use. Sodium bromide (NaBr), hydrogen chloride (HCl), sodium hydroxide (NaOH), 2,2,6,6-tetramethylpiperidin-1-oxyl (TEMPO), 10–15% sodium hypochlorite solution, 2,2-diphenyl-1-picrylhydrazyl (DPPH), calcofluor white and Nile red were all of reagent grade and purchased from Sigma-Aldrich (St Louis, MO, USA). Medium-chain triglycerides (MCT) oil was purchased from Amazon (https://www.amazon.com/Sports-Research-Keto-Organic-Coconuts/dp/B00XM0Y9SE/ref=sr_1_6?crid=2IYSLJED4R1Q4&keywords=mct%2Boil&qid=1673408530&sprefix=mct%2Boil%2Caps%2C224&sr=8-6&th=1 (accessed on 7 January 2023)) and used without further purification.

### 2.2. T-ChNCs Preparation

T-ChNCs were prepared using a TEMPO/NaBr/NaClO oxidation system with minor modifications based on a prior investigation [22]. Then, 2 g of chitin was suspended in 200 mL of water containing 0.032 g of TEMPO and 0.2 g of NaBr. NaClO was added to the suspension at a concentration of 10 mmol per gram of chitin. The pH of the slurry was maintained at 10.8 at 25 °C by continuously adding NaOH (0.5 M) during the course. Typically, the oxidation reaction could be completed in ~2 h, when no further consumption of the alkali was observed; after that, 2 mL of ethanol was added to quench the reaction, followed by adjusting the suspension pH to 7 using HCl (0.5 M). The suspension was then centrifuged at 12,000 rpm for 10 min and the supernatant was discarded. The TEMPO-oxidized chitin was thoroughly washed with distilled water. Residual ions were removed by dialyzing the T-ChNC precipitant against deionized water (DI water) for 48 h; after that, the solution was stored at 4 °C for further analysis.

### 2.3. O/W Pickering Emulsion Preparation

The T-ChNCs dispersion was diluted with DI water accordingly. One gram of MCT oil containing 1 mg mL^−1^ quercetin was added into 9 g T-ChNCs suspension and mixed by vortex, followed by sonication at 40% amplitude for 1 min in a cold-water bath. The final pH of the emulsion was around 7.

### 2.4. Characterization of T-ChNCs and Pickering Emulsions

*FTIR spectroscopy.* T-ChNCs were first precipitated from aqueous suspensions by using HCl (0.1 M), followed by a thorough wash with DI water. The precipitant was then freeze-dried overnight and the dried ChNC powder was measured using a Jasco FTIR 4100 spectrometer (Jasco Inc., Easton, MD, USA) under attenuated total reflection (ATR) mode at room temperature. All spectra were collected between 550 and 4000 cm^−1^ with a resolution of 2 cm^–1^.

*Dynamic light scattering and electrophoretic light scattering.* Particle sizes, including hydrodynamic size of T-ChNC particles and that of Pickering emulsion droplets, were determined by dynamic light scattering (DLS). The zeta potential of T-ChNCs and emulsion droplets was detected using electrophoretic light scattering (ELS) (Zetasizer Ultra, Malvern) at room temperature [23]. The measured electrophoretic mobility was used to obtain zeta potential via the Smuluchowski equation. The samples were characterized by diluting the nanocrystal suspensions or Pickering emulsion 500 times in DI water. The experiments were repeated three times and the mean values and standard deviations were obtained.

*Small-angle X-ray scattering.* Cross-sectional sizes of ChNC particles were determined by using small-angle X-ray scattering (SAXS) (XEUSS 2.0, Xenocs, Grenoble, France). In detail, the X-ray wavelength was 1.54 Å; the beam size was 0.5 × 0.5 mm and the sample-to-detector distance (SDD) was 1756 mm. SAXS measurements were performed for both dilute ChNC suspension with a concentration of 0.2 % (mass fraction) and DI water, loaded in a flow cell (quartz) with a diameter of 1.5 mm, using the same instrumental configurations. Scattering of the DI water served as the background and was subtracted from that of the suspension. Scattering data was presented in 1D profile, as the scattered intensity as a function of scattering vector, *q*, defined as *q =* (*4π/λ*)sin(*θ/2*), with *λ* and *θ* being the X-ray wavelength and the scattering angle, respectively. The scattering data were fitted against parallelepiped model [24] characterized by cross-sectional thickness *a* and width *b*.

*Scanning electron microscope.* The surface morphology of T-ChNCs was examined with SEM (Tescan XEIA FEG SEM, Brno, Czechia). The samples were freeze-dried overnight and then sputter-coated with gold for 70 s. The SEM micrographs were acquired at 10.0 kV and a working distance of 5.3 mm.

*Optical microscope.* Optical micrographs of T-ChNC stabilized O/W emulsions were captured using an optical microscope (Olympus, Tokyo, Japan) with a 50× lens.

*Confocal laser scanning microscope.* A 10% (mass fraction) O/W emulsion was prepared and double stained with Nile red and calcofluor white. Images were acquired using a Zeiss LSM 980 Airyscan 2 confocal laser scanning microscope (CLSM, Zeiss, Oberkochen, Germany) with a 20× lens. The dyed sample was placed on a microscope slide and covered with a glass coverslip at room temperature. The excitation and emission spectrum for Nile red and calcofluor white were 561 and 405 nm, respectively.

### 2.5. Emulsion Stability

Accelerated stability test was conducted by a centrifugation method [25]. Briefly, 5 mL of emulsion was centrifuged at 4000 rpm for 5 min in a plastic centrifuge tube at ambient temperature. Centrifugation speeds up phase separation, resulting in a layer of floated oil at the top, a creamy emulsion phase in the middle and a clear water phase at the bottom. A digital caliper was used to estimate the volumes based on their thickness. These volumes were used to calculate the cream fraction (volume of the remaining emulsion after centrifugation/total volume of the emulsion) and oil fraction (volume of MCT oil/total volume of the emulsion).

To evaluate storage stability, freshly fabricated samples were placed into graduated tubes and kept at room temperature or in a refrigerator at ~4 °C, sealed to prevent evaporation. After 0, 1, 3, 7 and 14 days of storage, the stability of the emulsions was examined via visual inspection and was assessed by droplet size and zeta potential. As methods in previous reports [26], to avoid disturbing the samples, we did not mix them before the test.

### 2.6. Encapsulation Efficiency (EE)

The EE was calculated after concentration determination of free unbounded quercetin as follows. Centrifuging took place at 4000 rpm for 5 min at room temperature. The supernatant was collected and determined by a UV-Vis spectroscopy (DU 730 UV/Vis Spectrophotometer, Beckman Coulter Inc., Brea, CA, USA) at λ = 372 nm with a standard curve (y = 0.0878x − 0.0589) of R^2^ = 0.9994 to calculate the solubility of quercetin. Afterward, encapsulation efficiency (EE) was calculated by the following formula:

(1) $$EE=\left(W_{Total}-W_{Free}\right)\;/\;W_{Total}\times 100$$

where *W_Total_* is the total quercetin weight in Pickering emulsion; *W_Free_* is the weight of free quercetin.

### 2.7. Radical Scavenging Assay

Antioxidant activity of Pickering emulsions with quercetin was performed by the DPPH radical scavenging method [27]. One Mole ethanolic DPPH solution was prepared and 0.5 mL was added to 3 mL of ethanol with 0.5 mL of Pickering emulsion samples, followed by shaking for 3 min and incubation in the dark at room temperature for 30 min. After passing through a 0.22 μm syringe filter, absorbance values were measured at 517 nm by the UV-Vis spectrophotometer. The antioxidant activity was calculated using the following equation:

(2) $$\%\mathrm{DPPH}\;\mathrm{scavenging}=\frac{A_{blank}-A_{emulsion}}{A_{blank}}$$

where *A_emulsion_* represents the absorbance of the DPPH radical in the presence of the Pickering emulsions and *A_blank_* refers to the absorbance of the DPPH radical alone.

### 2.8. In Vitro Release Study

The quercetin-release profiles from Pickering emulsion were obtained by a dialysis method [28]. A mixture of deionized water and ethanol (35:65 *v*/*v*) was used as the release medium. 2 mL of pure quercetin ethanol solution and quercetin-encapsulated Pickering emulsion were filled in the dialysis membranes (10 kDa), which were immersed in 100 mL of release medium and incubated at 37 °C with stirring (100 rpm). Periodically, 3 mL of release medium was withdrawn and replaced with an equal volume of fresh medium. The amount of quercetin released into the medium was measured at 372 nm using the UV-Vis spectrophotometer.

### 2.9. Statistical Analysis

All experiments were performed in triplicate and data were represented as mean ± standard derivation. All analyses were statistically tested for significance with one-way ANOVA using SAS software (Cary, NC, USA) and *p* < 0.05 was considered statically significant.

## 3. Results and Discussion

### 3.1. Characterization of T-ChNCs

**Figure 1:**
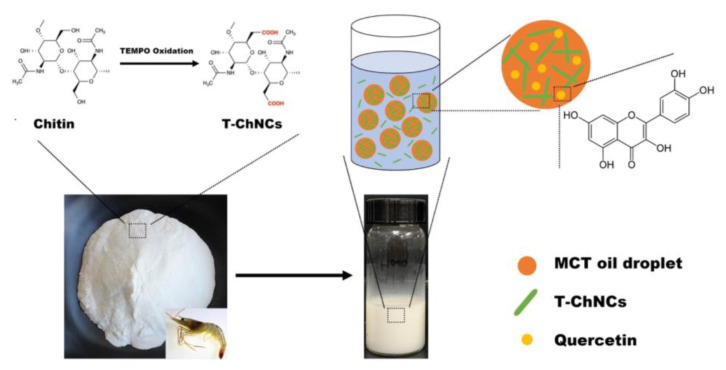
The scheme of chitin powder extracted from shrimp shells and modified by TEMPO oxidation for stabilization of Pickering emulsions and as quercetin carriers.

Figure 1 schematically illustrated the preparation of T-ChNCs. The shrimp shells were purified by removing proteins, minerals and other substances to obtain crude chitin powders. Subsequently, T-ChNCs were prepared by TEMPO oxidation. In this process, the hydroxyl group at the C6 position of chitin was oxidized to a carboxylate group, which leads to a negative surface charge of T-ChNCs and further contributes to the improvement of T-ChNCs’ water solubility. After COOH modification, T-ChNCs became amphipathic with appropriate surface charge to stabilize Pickering emulsion was used as stabilizer at the O/W interface. To comprehensively characterize the system, quercetin was encapsulated into the system.

**Figure 2:**
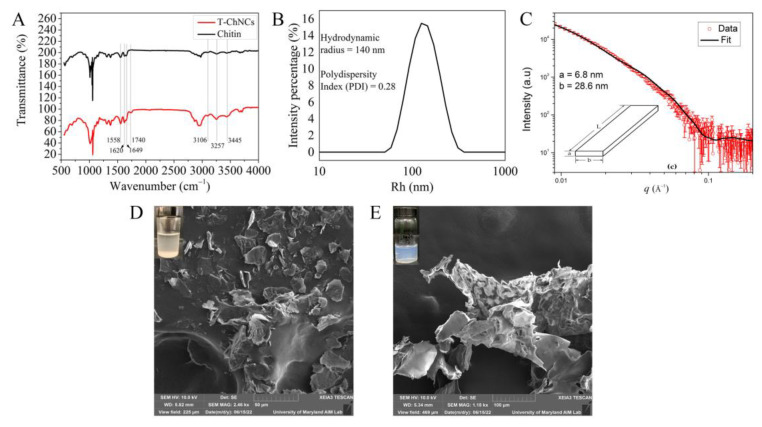
(**A**) FTIR spectra of chitin and TEMPO-oxidized chitin nanocrystals (T-ChNCs). (**B**) DLS analysis of T-ChNCs. (**C**) SAXS analysis of T-ChNCs. (**D**) SEM of chitin and (**E**) T-ChNCs.

The oxidation of chitin was verified by FTIR. Figure 2A shows the FTIR spectrum of original chitin powder and T-ChNCs. Both spectra had characteristic chitin functional groups, including an OH stretching vibration band at 3445 cm^−1^, NH stretching vibration band at 3257 and 3106 cm^−1^, an amide C=O stretching with hydrogen bonding with –NH and –OH groups at 1649 and 1620 cm^−1^, respectively and an amide C-N stretching and N-H bending at 1558 cm^−1^ [29,30]. After TEMPO oxidation, the sodium carboxylate groups in the products were converted to free carboxyl groups under acidic conditions and a slight absorption band, derived from the carboxylic groups, appeared at approximately 1740 cm^−1^, indicating that the chitin hydroxyl groups were successfully oxidized. This result agreed well with previous studies [31,32].

As shown in Figure 2B, the Z-Average size of T-ChNC was 139.8 ± 5.7 nm and the PDI was 0.28, indicating that the size of T-ChNC was relatively uniform. Considering that the true size of nanoparticles measured by DLS is not the real size of nanoparticles, but the hydrodynamic diameter of nanoparticles in solution, we further characterized T-ChNCs by SAXS. It can be seen from Figure 2C that T-ChNC was rod-like in shape with a cross-sectional thickness *a* of 6.8 ± 0.1 nm and width *b* of 28.9 ± 0.9 nm. The length L was much greater than a or b and was beyond the detector limit of SAXS, as revealed by the DLS data (279.7 ± 11.5 nm) [33]. The smaller size of T-ChNCs compared to other studies may be attributed to the increase in negative charge resulting in better dispersion and thus reduced particle size [34]. To test this hypothesis, the zeta potential of T-ChNCs was measured in a neutral environment (pH 7). Herein, T-ChNCs carried an intensive negative charge of −56.1 ± 1.6 mV. This indicated that the oxidation was sufficient and the chitin contained numerous carboxyl groups.

The morphology of chitin powder and T-ChNCs were significantly different (Figure 2D,E). The chitin powder was not soluble in water and deposited on the bottom, while the suspension of T-ChNCs was transparent with a light blue color due to light scattering from the nanoscale crystals [35]. The SEM image also revealed that chitin powder had a uniform appearance with smooth and oriented surfaces, while the surface of T-ChNCs was flocculent. This may be attributed to the strong positive charges on the surface of the T-ChNCs.

### 3.2. Factors Affecting Pickering Emulsions Stabilized by T-ChNCs

**Figure 3:**
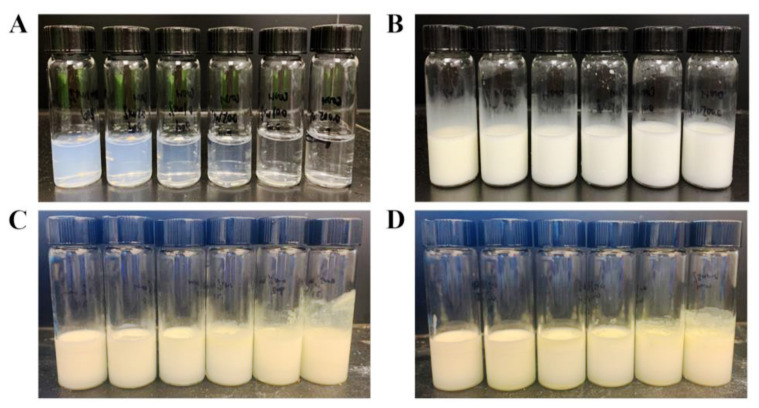
Photographs of (**A**) TEMPO-oxidized chitin nanocrystals (T-ChNCs) suspensions; Emulsions stabilized by T-ChNCs with different concentrations: (**B**) freshly prepared, (**C**) stored 14 days in the refrigerator, (**D**) stored 14 days at room temperature. Bottles from left to right contained 1.0, 0.5, 0.1, 0.05, 0.01 and 0.005 wt% of T-ChNCs.

The suspensions of T-ChNCs at different concentrations exhibited distinct appearances (Figure 3A). When the concentration of T-ChNCs was less than 0.01 wt%, the suspensions were clear and transparent. As the T-ChNCs content increased, the liquid gradually became turbid. As shown in Figure 3B, T-ChNCs enable the formation of emulsions as low as 0.005 wt%. The emulsions were formed as a typical non-transparent pseudoplastic fluid with no significant difference in appearance regardless of the content level of T-ChNCs. To identify critical factors on emulsions’ formation and stability, the droplet size and zeta potential were determined with variable preparation conditions. First, droplet sizes were in the range of 972.9 ± 86.0 to 1322.3 ± 447.7 nm when T-ChNC content varied from 0.005 wt% to 1.0 wt%. According to the optical microscopic image (Figure S1), it was evident that the density of the emulsion droplets was higher with higher T-ChNC concentrations, which may decrease creaming. A similar phenomenon was also reported in previous studies [36]. At high concentrations, sufficient T-ChNCs were attached to the interface of droplets and formed smaller droplets with a higher surface-to-volume ratio. These results were consistent with those reported in the literature [19]. According to the DLVO theory (named after Boris Derjaguin and Lev Landau, Evert Verwey and Theodoor Overbeek) [37], a higher absolute value of zeta potential can weaken or counteract the van der Waals force and avoid droplet aggregation, thus enhancing the physical stability of the emulsion [38]. In addition, emulsions formed with different T-ChNC contents under neutral pH had a zeta potential of around −42.6 ±1.0 mM.

As expected, T-ChNCs presented aggregation at pH 3 and prevented the formation of stable emulsions with large droplet sizes (12.8 ± 1.2 μm) and low zeta potential (−34.7 ± 1.0 mV, Figure S2A). Creaming was found immediately after preparing the emulsion (Figure S2B). The droplet size decreased from 12.8 ± 1.2 μm to 468 ± 71.3 nm with pH increased from 3 to 10. In alkaline conditions, the carboxyl group was deprotonated (-COO^−^) and carried a high negative charge (−45.9 ± 0.7 mV). This effectively prevented droplets from approaching each other and from aggregating into larger clusters.

### 3.3. Storage Stability

The storage stability of T-ChNC stabilized emulsions was carried out at two different conditions (4 °C and 25 °C). Studies have shown that increasing the concentration of emulsified particles decreases droplet size, resulting in a more stable Pickering emulsion [17,36]. Therefore, emulsions containing 0.005, 0.01, 0.05, 0.1, 0.5 and 1.0 wt% T-ChNCs were studied. Figure 3 displays images of freshly-prepared emulsions and storage in the refrigerator and at room temperature for 14 days. By visual observation, no significant separation was observed under either condition for all treatments. This indicated that emulsions stabilized by T-ChNCs exhibited superior stability when compared to bare chitin. No oil phase separation was observed after 14 days even with T-ChNC content as low as 0.005 wt%, demonstrating an excellent stability against coalescence and Ostwald ripening.

**Figure 4:**
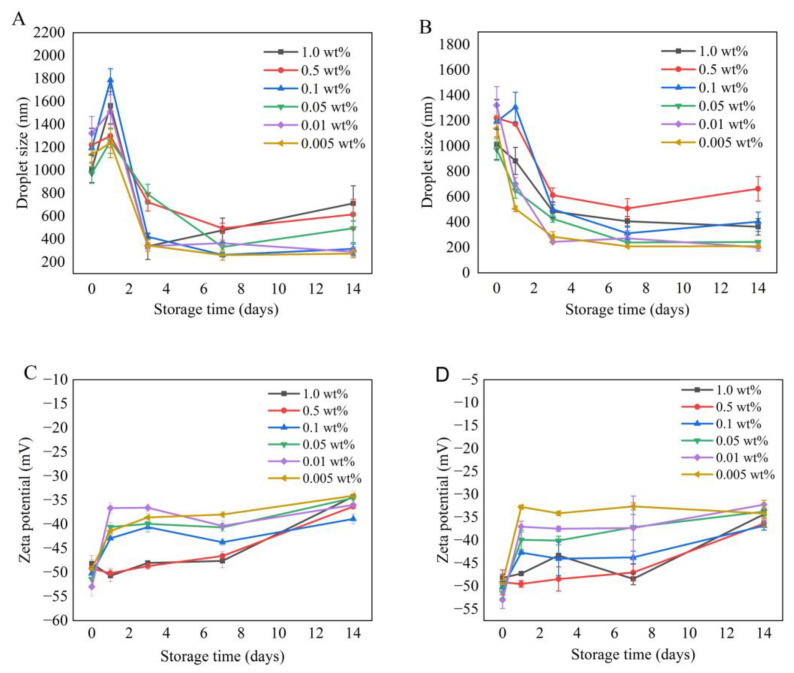
Effects of storage time and temperature on droplet size and zeta potential of emulsions with different concentrations of TEMPO-oxidized ChNCs at pH 7: (**A**) change in droplet size at 4 °C; (**B**) change in droplet size at 25 °C; (**C**) change in zeta potential at 4 °C; (**D**) change in zeta potential at 25 °C.

The droplet size and zeta potential of Pickering emulsions were measured and obtained during 14 days of storage (Figure 4). Droplet size showed a significant decrease after 14 days of storage in the refrigerator and at room temperature. This might be due to the thermal motion of the molecules and gravitational effects causing the deposition of larger particles, resulting in a decrease in the droplet size in the emulsion. This result was consistent with previous reports [39]. Similar results were obtained for the change in zeta potential under both storage conditions, with the absolute value of zeta potential decreasing as storage time was prolonged. Besides, it is also worth highlighting that the coalescence process was kinetically slower at 4 °C than at 25 °C; thus, the increase in droplet size could be observed as an indication of the coalescence process at a sampling frequency of 1 day. Then the droplet size decreased due to the sediment of the larger droplets. At 25 °C, on the contrary, the coalescence was accelerated and the initial step of increase in droplet size was not observed.

Accelerate phase separation in emulsions by centrifugation provided a rapid estimation of emulsion stability. Table S1 shows the data on emulsion stability at different concentrations of T-ChNCs. As T-ChNC content increased, the cream fractions increased, while the oil fractions decreased steadily. The reason may be an increase in effective droplet density due to the adsorption of T-ChNCs and a rise in viscosity at higher T-ChNCs concentrations [36]. After centrifugation, a thin layer of pure oil was observed floating on top of the liquid for emulsions formed by 0.005 wt% and 0.01 wt% of T-ChNCs. However, no oil floating was observed when the concentration of T-ChNCs was above 0.05 wt%, demonstrating the T-ChNCs effectively inhibited droplet coalescence. This might be caused by sufficient T-ChNCs to prevent droplets from attracting each other. Conversely, few T-ChNCs may not completely cover droplets, resulting in poor emulsion stability [21].

### 3.4. Microstructure of O/W Pickering Emulsions Stabilized with T-ChNCs

**Figure 5:**
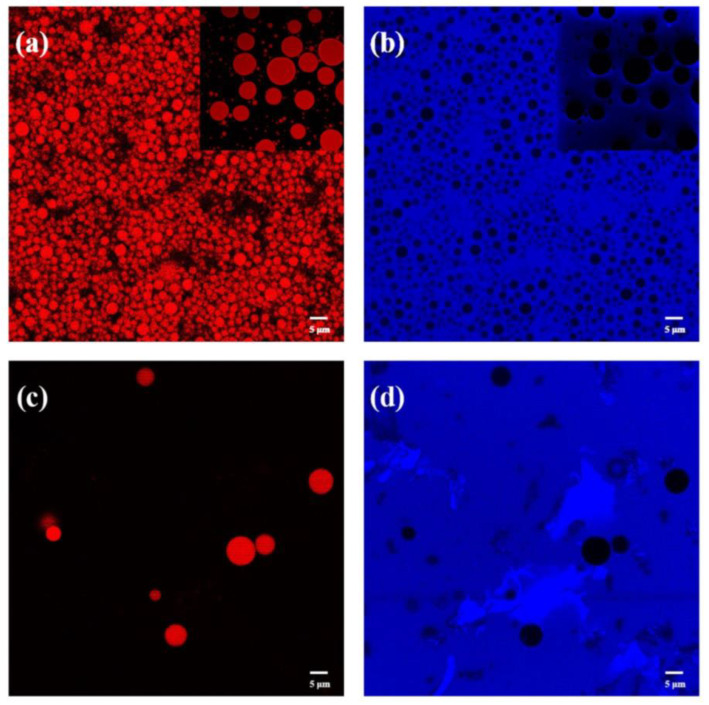
Confocal laser scanning microscopy images of fresh Pickering emulsion stabilized by 1.0 wt% TEMPO-oxidized ChNCs (T-ChNCs) (**a**,**b**) and chitin powder (**c**,**d**): MCT oil stained with the Nile red (**a**,**c**), T-ChNCs or chitin powder dyed by Calcofluor white strain (**b**,**d**). The volume fraction of MCT oil is 0.10. The scale bar is 5 μm.

The microstructure of Pickering emulsion was investigated by using CLSM (Figure 5). Herein, Figure 5a showed the oil phase labeled in red surrounded by unlabeled water, indicating an oil-in-water system. T-ChNCs were labeled blue (Figure 5b) and well dispersed in the water, resulting in the aqueous phase appearing as blue. The magnified image (small image in the upper right corner) clearly showed that the blue light shined more intensively around the oil droplets. Since the T-ChNCs were colored blue, it indicated that a large amount of T-ChNCs was aggregated at the O/W interface. Therefore, it can be identified as a Pickering emulsion. In comparison, the samples prepared by chitin powder were significantly different. The chitin powder was not uniformly distributed in the aqueous phase and the oil droplets were dispersed and sparse (Figure 5c,d). Two distinct images proved that T-ChNCs acted as stabilizers at the O/W interface and successfully formed the Pickering emulsion.

### 3.5. Encapsulation Efficiency

**Figure 6:**
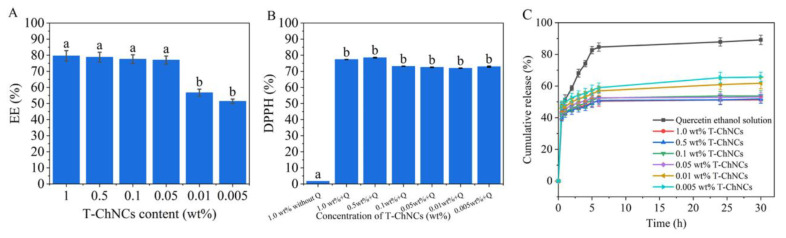
(**A**) EE of TEMPO-oxidized ChNCs (T-ChNCs); (**B**) Antioxidant activities of Pickering emulsion stabilized by T-ChNCs with different concentrations; (**C**) Release profiles of quercetin from Pickering emulsion stabilized by T-ChNCs with different concentrations. Different letters (a, b) represent significant differences (*p* < 0.05).

Pickering emulsions stabilized by T-ChNCs were applied for the encapsulation of quercetin. EE refers to the mass ratio of the encapsulated quercetin to the total amount of quercetin added during the preparation. The EE of the formulations obtained by varying the T-ChNCs content are presented in Figure 6A. The quantification of free quercetin in the supernatant showed a range of EE that varied between 51.4 ± 1.4% and 79.6 ± 3.2%. Among different T-ChNCs concentrations, 1.0 wt% of T-ChNCs showed the highest encapsulation efficiency of 79.6 ± 3.2%.

Additionally, DPPH radical scavenging activity demonstrated successful encapsulation of quercetin in Pickering emulsions. As shown in Figure 6B, T-ChNCs without quercetin exhibited no antioxidant activity, whereas Pickering emulsions containing quercetin displayed antioxidant activity, ranging from 71.9 ± 0.1% to 78.5 ± 0.2% with T-ChNCs content ranging from 0.005 wt% to 1.0 wt%.

The release of quercetin in vitro was studied by the dialysis bag method. As shown in Figure 6C, the behavior of quercetin released from Pickering emulsions was compared with the control group of quercetin ethanol solution. Quercetin ethanol solution exhibited a faster release rate with 82.7 ± 2.3% of quercetin being dissolved within the initial 5 h. In contrast, Pickering emulsions stabilized by different concentrations of T-ChNCs released only 49.3 ± 2.8 to 57.4 ± 3.2% of quercetin within the first 5 h, which is half of the pure quercetin in an ethanol solution. It is clear that the release of quercetin from the Pickering emulsions was significantly delayed and achieved slow release. From the release profiles, it is apparent that after 6 h, quercetin release reached a plateau. As the T-ChNC content increased, the release rate of quercetin in vitro became slower. The results indicated that adequate T-ChNCs wrapped the oil droplets containing quercetin so that they did not leak out easily. Thus, the stability of the Pickering emulsion was demonstrated.

## 4. Conclusions

In this paper, T-ChNCs were oxidized by chitin from marine waste shells and were explored as a potential material to stabilize Pickering emulsions. It was found that T-ChNCs as low as 0.005 wt% were able to form emulsions at pH 7. The emulsions remained stable after 14 days of storage at 4 °C and 25 °C. When the concentration of T-ChNCs was greater than 0.05 wt%, the emulsion was more stable which was due to the fact that no oil phase separation was observed after centrifugation. Within adjusted pH range of 3.0–10.0, emulsions with 0.1 wt% T-ChNCs are more stable at higher pH values with small droplets (468 ± 71.3 nm) and a high negative charge (−45.9 ± 0.7 mV). In addition, the synthesized Pickering emulsions were able to encapsulate up to 79.6 ± 3.2% of quercetin and in vitro release studies showed that Pickering emulsions retarded the release rate, which decreased to 49.3 ± 2.8–57.4 ± 3.2% within 5 h compared to the unencapsulated (82.7 ± 2.3%). The study provided an approach for preparing Pickering emulsions stabilized by T-ChNCs, which can effectively act as a promising carrier of quercetin for delivery applications.

## Data Availability

Data is contained within the article or Supplementary Material.

## Supplementary Materials

The following supporting information can be downloaded at: https://www.mdpi.com/article/10.3390/foods12020367/s1, Figure S1: Optical micrographs of fresh Pickering emulsions stabilized with different concentrations of TEMPO-oxidized ChNCs at pH 7 (The scale bar was 10 μm): (A) 1.0 wt%; (B) 0.5wt%; (C) 0.1wt%; (D) 0.05wt%; (E) 0.01wt%; (F) 0.005wt%. Figure S2: (A) Mean droplet diameter and zeta potential of Pickering emulsions stabilized by TEMPO-oxidized ChNCs with different pH values. (B) Photographs of fresh Pickering emulsion (a) at different pH levels (from left to right pH 3, 5, 7, 10) and (b) stored for 14 days. Table S1: Cream fraction and oil fraction of emulsions with different concentrations of T-ChNCs were added.
