# Supplementary material for: Development of Stable Pickering Emulsions with TEMPO-Oxidized Chitin Nanocrystals for Encapsulation of Quercetin
Source: Foods. 2023 Jan 12;12(2):367. doi: 10.3390/foods12020367 (PMC9857725; doi:10.3390/foods12020367)
Supplement: Supplementary file 1 [file foods-12-00367-s001.zip › foods-2068378-supplementary.pdf]

Supplementary Material

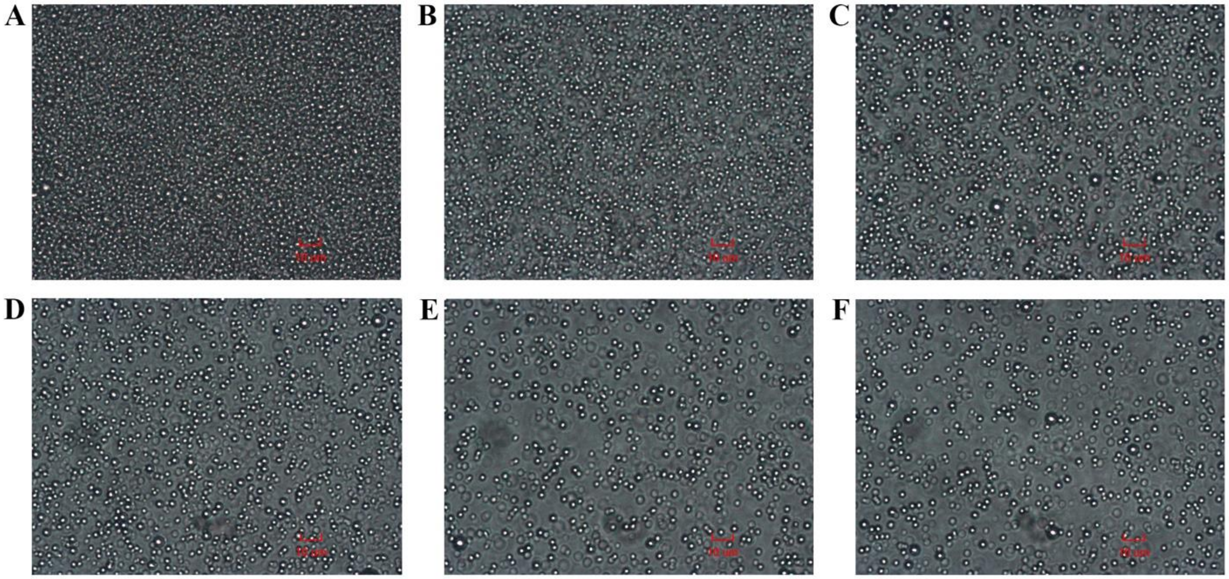

Figure S1. Optical micrographs of fresh Pickering emulsions stabilized with different concentrations of TEMPO-oxidized ChNCs at pH 7 (The scale bar was 10 µm): (**A**) 1.0 wt%; (**B**) 0.5wt%; (**C**) 0.1wt%; (**D**) 0.05wt%; (**E**) 0.01wt%; (**F**) 0.005wt%.

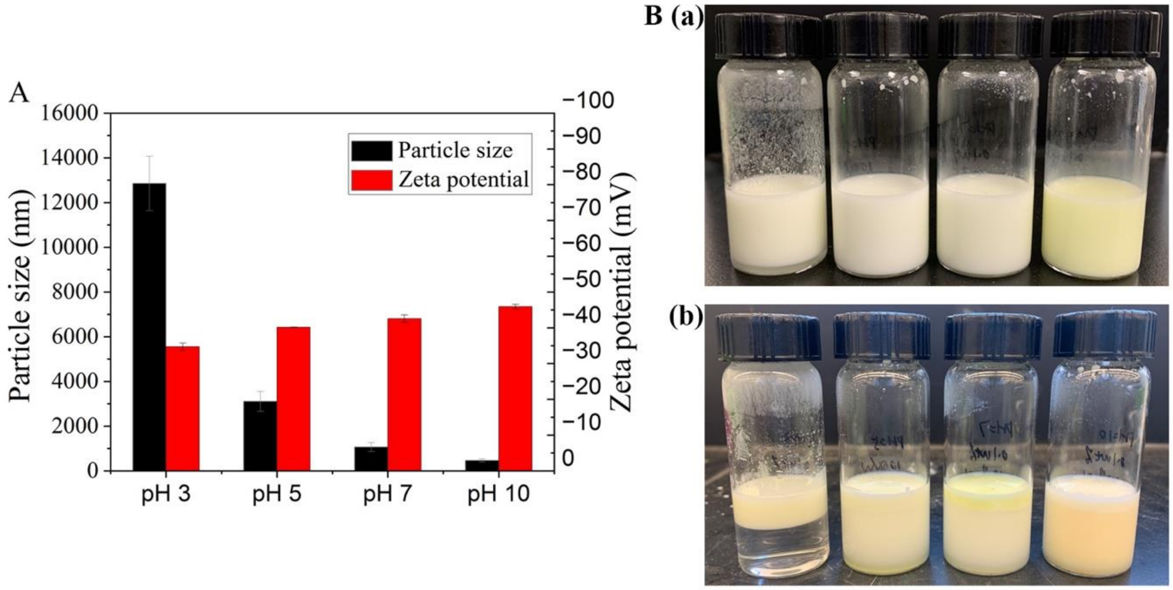

Figure S2. (**A**) Mean droplet diameter and zeta potential of Pickering emulsions stabilized by TEMPO-oxidized ChNCs with different pH values. (**B**) Photographs of fresh Pickering emulsion (**a**) at different pH levels (from left to right pH 3, 5, 7, 10) and (**b**) stored for 14 days.

Table S1. Cream fraction and oil fraction of emulsions with different concentrations of T-ChNCs were added.

**T-ChNCs concentration (wt%)**	**Cream fraction**	**Oil fraction**
1	0.14±0.00	0±0
0.5	0.14±0.01	0±0
0.1	0.13±0.01	0±0
0.05	0.12±0.01	0±0
0.01	0.10±0.01	0.03±0.01
0.005	0.10±0.01	0.68±0.01
